# Novel Bio-Based Materials and Applications in Antimicrobial Food Packaging: Recent Advances and Future Trends

**DOI:** 10.3390/ijms22189663

**Published:** 2021-09-07

**Authors:** Chunming Tan, Fei Han, Shiqi Zhang, Pinglan Li, Nan Shang

**Affiliations:** 1 Beijing Laboratory for Food Quality and Safety, College of Food Science and Nutritional Engineering, China Agricultural University, Beijing 100083, China; cmtan_ouc@163.com (C.T.); feihan@cau.edu.cn (F.H.); zsqkevin@163.com (S.Z.); 2 College of Engineering, China Agricultural University, Beijing 100083, China; 3 Department of Nutrition and Health, China Agricultural University, Beijing 100083, China

**Keywords:** bio-based materials, bio-nanocomposites, antimicrobial, food packaging, pathogens, spoilage microorganisms

## Abstract

Food microbial contamination not only poses the problems of food insecurity and economic loss, but also contributes to food waste, which is another global environmental problem. Therefore, effective packaging is a compelling obstacle for shielding food items from outside contaminants and maintaining its quality. Traditionally, food is packaged with plastic that is rarely recyclable, negatively impacting the environment. Bio-based materials have attracted widespread attention for food packaging applications since they are biodegradable, renewable, and have a low carbon footprint. They provide a great opportunity to reduce the extensive use of fossil fuels and develop food packaging materials with good properties, addressing environmental problems and contributing significantly to sustainable development. Presently, the developments in food chemistry, technology, and biotechnology have allowed us to fine-tune new methodologies useful for addressing major safety and environmental concerns regarding packaging materials. This review presents a comprehensive overview of the development and potential for application of new bio-based materials from different sources in antimicrobial food packaging, including carbohydrate (polysaccharide)-based materials, protein-based materials, lipid-based materials, antibacterial agents, and bio-based composites, which can solve the issues of both environmental impact and prevent foodborne pathogens and spoilage microorganisms. In addition, future trends are discussed, as well as the antimicrobial compounds incorporated in packaging materials such as nanoparticles (NPs), nanofillers (NFs), and bio-nanocomposites.

## 1. Introduction

**Figure 1:**
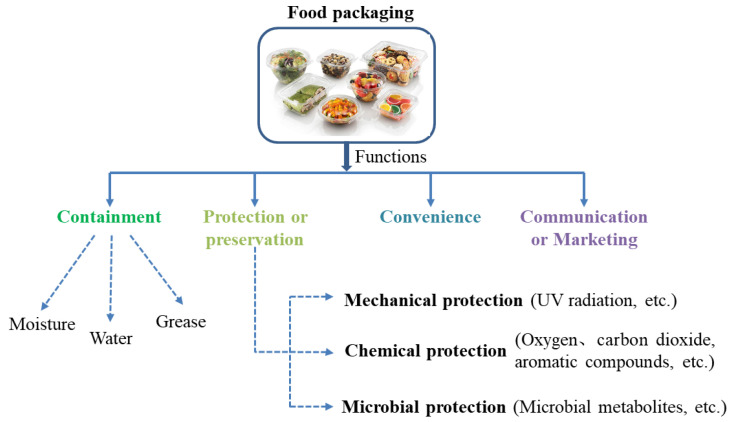
The functions and properties of packaging system.

The essential functions of packaging are coverage and conservation of foodstuffs, retaining the food safety, quality, and increasing the shelf life of food products during transport, storage, and marketing. It decreases post contamination and protects them from physical, chemical, environmental, and microbial hazards [1,2] (Figure 1). Packaging also provides information regarding products, simplifies their end-use suitability and communication (marketability and acceptability), and facilitates the food control process from distribution to the consumer’s table [3,4]. Food contamination not only creates the problems of food insecurity and economic loss, but also contributes to food waste, which is another global environmental problem [5]. Furthermore, foodborne pathogens denote a major threat to public health; it is a critical reason for illnesses, hospitalizations, and deaths of people. In the United States (2018), the number of cases of foodborne illness was 35,027; outbreaks are frequently reported every month with the number of 204 in April [6]. Al-Tayyar et al. [7] reported that the United States foodborne illness cost is approximately 152 billion dollars annually for long term health and immediate medical care, among which ¼ of expenditures are caused due to foodborne illness linked with the use of processed, canned, and fresh products. The food industries and retailers have to face product degradation or loss and pay millions of dollars as microbiological contamination costs. Tamayo et al. [8] reported that *Staphylococcus aureus*, *Listeria monocytogenes*, *Toxoplasma gondii*, *Campylobacter* spp., and *Salmonella* spp. are the widely spread pathogens; they cause illness, hospitalizations, or even deaths among human beings when contamination of food is caused. In addition, food spoilage is another troubling issue faced by the food industry, as it negatively affects the food quality and it could lead to huge economic losses for producers, retailers, and consumers [9]. The cross contamination of spoilage microorganisms on raw materials or processed foodstuffs can result in changes of nutritional and sensory characteristics of food. It causes approximately 1.3 billion tons of food waste every year, and the heavy expenses in medical care account for more than 77.1 billion dollars annually, only in the USA [10,11]. The microbial contamination of foods can occur anywhere when food is kept in an external environment, such as stages of slaughtering, distribution, shipping, storage, and retail display [7]. Therefore, effective packaging is a compelling obstacle for shielding food items from outside contaminants and can maintain its quality. 

There are diverse materials applicable for packaging, among them plastics for food packaging are the most and reached 129 million tons globally in 2015, and was expected to reach USD 262 billion by 2020 [2,12,13]. Plastics have been extracted from non-renewable resources of fossil fuels since the middle of the twentieth century and they face difficulty in recycling and disposal [2,14]. Due to organic materials being contaminated and present on food packaging plastics, thus plastic recycling is not suitable for food packaging systems [15]. Sharma et al. [2] indicated approximately 4.8–12.7 million tons of plastics were thrown in the ocean during the year of 2010, and more than 30% of the waste produced by plastic processing was landfilled in the year 2014. Therefore, the use of non-biodegradable and non-renewable materials (e.g., cans, plastics, glass bottles, paper bags, and metals) in packaging applications has caused numerous environmental concerns about their decomposition and overexploitation of natural resources and thus there is demand for seeking a suitable alternative for traditional packaging materials [4,16]. Biopolymers have developed widespread attention for food packaging applications, since they are biodegradable, renewable, and have a low carbon footprint [14,17]. They provide a great opportunity to reduce the extensive use of fossil fuels and develop biobased food packaging materials [18], addressing the environmental problems and contribute significantly to the sustainable development [19]. Studies have reported that the application of biopolymer-based materials can reduce packaging waste and solve the waste disposal problems to a certain extent [16,20] and have the potential to be an alternative for synthetic plastic packaging materials, especially for single-use and short-lived food packaging purposes [2,17,21]. Abdul Khalil et al. [16] indicated that bio-based materials successfully address concerns regarding cost, energy consumption, recycling procedure, and sustainability when compared to synthetic plastics. At present, various bio-based polymers have been used in food packaging materials, including polysaccharide-, protein-, and lipid-based materials [2,7]. Among then, coatings are provided via biopolymers which can be applied directly in a liquid form on foodstuffs via immersion, spraying, and brushing, etc. [22], and the carbohydrate and protein-based packaging films have good mechanical characteristics and are generally considered as effective oxygen hinderers below intermediate humidity, but their physical features (like water vapor barrier), mechanical properties, and antimicrobial activity are still poor; thus, they are hard to use in industries [1,7]. Therefore, the combination of polysaccharides with other polymers offers a new way for the development of various tailor-made packaging and coating materials for medical and food purposes [3,23]. 

However, compared with petroleum-based materials, weak mechanical and barrier properties of biopolymer-based packaging materials limited their widespread application in industry [16,18]. Therefore, greater efforts are underway to improve the properties of bioplastics to make them suitable and successful for practical use in food packaging. Abdul Khalil et al. [16] reported that various methods have been proposed for this purpose, such as addition of plasticizer and nanomaterials, chemical modification of polymer, treatment of gamma irradiation, etc. In recent years, biopolymer-based nanocomposite packaging materials have been widely considered due to their good mechanical, thermal, chemical resistance, biodegradable, antimicrobial, and gas barrier properties [2]. Bio-nanocomposites are novel materials with two or more phases (continuous and discontinuous), and the nanoparticles can interact with other matter at the atomic, molecular, or macromolecular level thus changing functional properties. The continuous phase is biopolymers such as polysaccharides, proteins, lipids, nucleic acids, etc., while the discontinuous phase is filler such as silicates, carbon nanotubes, silver/silver oxide (Ag/AgO), zinc oxide (ZnO), titanium dioxide (TiO_2_), etc. [17]. Among the metal oxides, ZnO is one of the most broadly utilized materials in different fields because of its antimicrobial activity and photocatalytic properties, such as zinc oxide nanoparticles (ZnO-NPs) and ZnO-SiO_2_ nanocomposites [24]. These nanoparticles are also biocompatible with cells so they can be used as a defense against food pathogens [7,25]. 

Antimicrobial packaging is a robust technology to provide fresher, safer, and higher quality food products, and some new technologies have been studied which are associated with foodborne pathogenic and spoilage microorganisms. It is a form of active packaging (including oxygen scavengers, moisture absorbers, ultraviolet barriers, and other mechanisms delivering antioxidant, flavoring, or antimicrobial activity), and the packaging material interacts with the packaged food in a desirable way [11,26]. Antimicrobial packaging stands out as an emerging technology that not only prolongs the shelf life of food products but also helps maintain their quality [27]. The antimicrobial active packaging forms including directly fixing the antibacterial agents into a polymer matrix, using the inherently antimicrobial activity of polymers, coating it onto the packaging surface or immobilizing it in pouches and pads via ion or covalent linkages [28]. Many types of antibacterial agents that are used in food packaging include natural antibacterial agents (e.g., chitosan, nisin, lysozyme, plant essential oils, tea polyphenols, etc.), organic synthetic antibacterial agents (such as chloramphenicol and nalidixic acid), and inorganic antibacterial agents (some of the oxidized nanoparticles) [29,30,31,32]. At present, many inorganic and metal nanoparticles have been implemented to synthesize active food packaging materials and to extend the shelf life of foodstuffs [33]. However, it is noteworthy that the compatibility between nanomaterials and polymer matrices is considered the biggest challenge for the preparation of bio-nanocomposites. In this review, we have evaluated recent works and applications of bio-based materials from different sources in the food packaging sector along with their antimicrobial properties and mechanisms. Applications and future trends of packaging materials are also discussed, as well as the antimicrobial compounds incorporated in packaging materials. This review provides interesting information for the food industry about new packaging materials that could be considered as renewable and sustainable.

## 2. Types of Bio-Based Materials in Antimicrobial Food Packing

**Figure 2:**
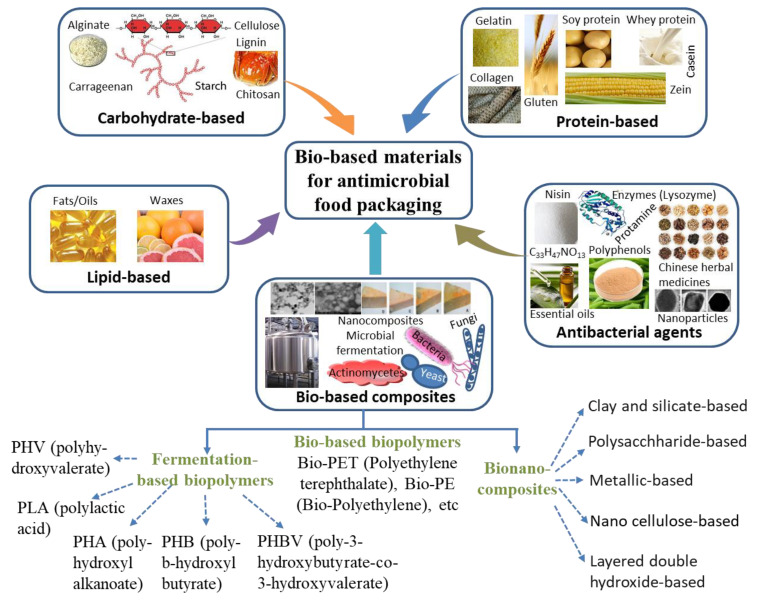
Types of bio-based materials used for antimicrobial food packaging application.

Bio-based materials are eco-friendly sustainable packaging materials which are used to create an obstacle for microorganisms (pathogens and spoilage bacteria) or insects, as a result of which spoilage and disease are completely prevented [7]. Bio-polymers are the most important source of sustainable materials at the industrial scale and have been utilized in industries due to their attractive properties, such as biocompatibility, chemical stability, and biodegradability [16]. Generally, the types of bio-based antimicrobial materials in food packing include carbohydrate (polysaccharide)-based materials, protein-based materials, lipid-based materials, antibacterial agents, and bio-based composites (Figure 2).

### 2.1. Carbohydrate-Based Materials

Polysaccharides like chitosan, starch, and cellulose are biodegradable and non-toxic, some of them with a semi-crystalline state and acid hydrolysis properties have been found to be potential sources of nanosized reinforcements since they can release crystalline sections [19]. Chitosan (CS) is one of the most extensively studied and used biopolymers in food coating and packaging and has excellent properties of film forming, antimicrobility, and biodegradability [3,24,34]. Antibacterial activity of chitosan depends on pH value, molecular weight, and degree of deacetylation, and its casted film has high antibacterial activity at lower pH due to the fact that the amino group is in a protonated form [35,36]. Chitosan can be modified to improve the antibacterial activity; for example, an edible film based on chitosan (2–3%, *w*/*v*) and gamma-aminobutyric acid-rich fermented soy protein showed great antimicrobial property [37]. In recent years, chitosan and its derivatives have been strongly exploited as an alternative natural antibacterial and antioxidant agent [38,39], with the forms including powder [40], coating [41], casted film [42], and nanoparticles [43,44,45]. Currently, chitosan polysaccharide or its derivatives are attracting extensive scientific testing for application in various fields [46]. It is also widely considered as an excellent material for nanofibers production due to the film forming ability, safety, and large antibacterial property [47]. 

Starch, another widely explored naturally renewable carbohydrate polymer (extracted from rice, maize, corn, wheat, barley, potato, vegetables, soya, etc.), contains amylose (linear molecules linked to each other through α-1, 4 glycosidic bond) and amylopectin (branched chain molecule with branches occurring at α-1, 6 bonds) [19]. They are mainly used as raw material in the production of biodegradable films or bio-plastics due to their non-toxic, biodegradable features, low cost, plasticity, and ready accessibility for industries [48]. The starch-based films commonly possess high properties of oxygen barrier, but they have poor properties of moisture barrier, while the addition of chitosan in starch can change the sensitivity of water and enhance the mechanical and barrier features of starch films [49]. Starch and its derivatives are the most general kind that have been explored to prepare bio-nanocomposite materials for food packaging applications [50], and the cheaper food spoilage detecting packaging materials can be produced using a conducting biodegradable polymer material. However, more research is required on starch that focuses on decreasing retrogradation and reducing water absorption of the material to maintain stiffness and mechanical strength during storage, making the food packaging material simpler, cheaper, more active, yet smarter, where the consumer is able to assess the quality, safety, shelf life, and nutritional values of the contents of packet. 

In addition, polysaccharides such as chitin and cellulose have been found to be potential sources of nanosized reinforcements. Cellulose is a linear homo-polysaccharide composed of β-d-glucopyranose units connected by β-1–4-linkages with a repeating unit of cellobiose, including crystalline and amorphous domains [51]. Siqueira et al. [52] reported the structure of cellulosic fibers is composed of cellulose, lignin, and hemicellulose; ash and extractives are also present in varying amounts depending on their origin. Cellulose and cellulose derivatives (e.g., carboxymethyl cellulose, cellulose acetate, and ethyl cellulose) can be used in the preparation of cellulose-based films for food packaging, the most commonly used form is cellophane [16], and the cellulose fibrils embedded in a learning matrix or extracted from a wide range of cellulose rich sources can obtain cellulosic nanofibers [51,53]. Recently, nanocellulosic materials have attracted considerable attention from scientists for improving the mechanical, thermal, antimicrobial, and barrier properties of packaging materials while retaining the biodegradable and non-toxic characteristics [16,54]. These nanofibers have wide availability since they may provide superior rigidity and tensile, flexural, and thermal properties and are recognized as eco-friendly material for various food packaging (such as fresh produce, dried foods, confectionary, home personal products, dairy, meat, and pouches) due to their security, recyclability, and reusability [55,56]. Furthermore, the food packaging materials prepared using oxidized cellulose nanocrystals and cyclodextrin enable antibacterial molecules to be loaded and released over a prolonged period of time [57]. However, the use of nanocellulosic materials is limited at commercial level when compared to petroleum-based plastic materials [58]. Future studies are recommended to focus on innovative approaches with cellulose nanofibers to design nanocellulosic materials for sustainable packaging, thus providing a better experience for the end user, and efficient manufacturing systems also need to be established. 

Furthermore, alginate, carrageenan, and agar are generally used as hydrocolloids for edible films or coatings. Alginate is an attractive component for film formation due to its non-toxic nature, biocompatibility and stabilizing, biodegradability, low cost, gel-producing, and thickening properties [59]. The edible films and coatings fabricated using carrageenan have already been utilized in various fields of the food industry such as fresh and frozen meat, poultry, and fish due to their good gas barrier properties [60]. 

### 2.2. Protein-Based Materials

As for protein-based packaging (e.g., whey protein, soy protein, wheat gluten, zein, casein, collagen, gelatin-based, etc.), they have been comprehensively investigated regarding their thermal, mechanical, and barrier properties more than any other bio-based packaging material due to their extensive resources, biodegradability, availability, and release control of additives and bioactive compounds (antimicrobial agents) into the packaging system, such as edible packaging (films and coatings) [4,61]. For example, whey proteins isolates (WPI) can form flexible, transparent films with a low mechanical strength and high water vapor permeability due to the fact that they have a high amount of hydrophilic amino acids in their structure, so they have better barrier properties than polysaccharide or lipid-based films [62]. Gelatin is also an appropriate protein-based polymer for the fabrication of biodegradable packaging because of its good properties (abundance, low cost, biodegradable, functional agent carrier, oxygen barrier, and excellent film forming activity), and it has been successfully and widely deployed to create packaging films [63,64]. Lin et al. [65] developed a bio-nanocomposite for food packaging via embedding gelatin nanofibers into moringa oil/chitosan nanoparticles (CS-NPs) which exhibited high antibacterial activity against *L. monocytogenes* and *S. aureus* on cheese. It is noteworthy that the major approach of obtaining protein films is via solution casting, which includes solubilization of protein in an appropriate solvent and then casting and vaporizing of solution on a non-sticky surface. However, protein coatings can be utilized mainly by dipping and spraying and in some cases brushing of a food product [4,66]. Protein-based packaging could be carriers of antimicrobials, antioxidants, nutrients, colorants, and flavoring agents, generally used in cheese, fruits and vegetables, meat products, and fishery products to boost their durability and acceptability [67]. 

Protein-based films have good mechanical properties and are generally considered as effective oxygen hinderers below intermediate humidity, but their physical features (such as water vapor barrier, tensile strength, and thermal durability) are still poor and thus difficult for use in industries [1,7]. Therefore, certain approaches have been presented to eliminate the limitations of protein-based films, including cross-linking (physical, chemical, and enzymatic), blending with other biopolymers, and reinforcing with nanoparticles [61,68,69]. Nanotechnology proved to be a potential approach for significantly improving protein-based films, and various investigations have been carried out on this matter. Zubair and Ullah [17] pointed out that the most studied protein-based bio-nanocomposites suitable for food packaging are soy protein isolates, gelatin proteins, corn zein, and wheat gluten proteins. However, protein-based bio-nanocomposites are still in their early stages and more research should be considered in the future. Furthermore, in order to commercially produce protein films, future studies are recommended to focus on the production of these films via extrusion processes rather than solution casting at laboratory scales. It encourages the scientists to develop novel preservation strategies by incorporating bioactive agents (such as bioactive reinforcements with antioxidant capacity and bio-based reinforcements with antimicrobial capacity). 

### 2.3. Lipid-Based Materials

Lipids are good components of edible films and coatings for food applications, including animal and vegetable oils and fats (such as lard, butter, fatty acids, extracts, and mono-, di- and triglycerides, etc.), waxes (paraffin, carnauba, beeswax, candelilla, and jojoba), natural resins (chicle, guarana, and olibanum), and emulsifiers and surface-active agents (fatty alcohols, lecithin, and fatty acids) [60,70]. Researchers have investigated the application of lipid incorporation into edible film coating, and the hydrophobicity, cohesiveness, flexibility, and moisture barriers are improved, leading to prolongation of quality and inhibition of microorganisms in fresh and processed meats [71]. Films composed of lipids have good water vapor barrier properties but exhibit reduced mechanical strength and increased oxygen permeability. However, the combination with hydrophilic materials or lamination with a hydrocolloid film lipid layer can improve the mechanical properties. For example, Jimenez et al. [72] studied the effects of saturated and unsaturated fatty acids on hydroxypropyl-methylcellulose based films and found that fatty acids can form stable layers in film matrices and improve the moisture barrier properties. Furthermore, reinforcing with nanoparticles proved to be an effective method for significantly improving lipid-based film properties [73]. 

### 2.4. Antibacterial Agents

Many types of antibacterial agents have been used in food packaging including natural antibacterial agents (e.g., bacteriocins, ε-polylysine, enzymes, plant essential oils, tea polyphenols, etc.), inorganic antibacterial agents (some of the oxidized nanoparticles and metals), and organic synthetic antibacterial agents [29,30,31,32]. Natural antibacterial agents are extracted and purified from animals, plants, and microorganisms, and they are generally considered to be safe, healthy, and eco-friendly antibacterial agents [74,75]. For instance, the natural antimicrobial peptides such as nisin, natamycin, leucocin, enterocin, and pediocin are recognized as bio-preservatives that can be used for inhibiting or killing pathogens or spoilage bacteria which can cause food spoilage [76]. Recently, essential oils (EOs) have been the subject of numerous research studies, due to the broad-spectrum antibacterial activity, and applied as a sustained release preparation in food packaging [31,77]. According to the investigations of Ju et al. [30] and Khaneghah et al. [32], the hydrophobicity of EOs and their constituents is an important characteristic, which allows EOs to interact with the lipids of the microbial cell membrane and mitochondria, making the structures less organized and more permeable, thus ions and other cell contents outflow. However, it should be noted that EOs are a complex mixture of compounds with main components that include alkaloids, flavonoids, isoflavones, monoterpenes, phenolic acids, carotenoids, and aldehydes [31]. Different chemical components can act through different mechanisms, and the same chemical composition may also have different effects when applied to different types of microorganisms. Furthermore, EOs are nearly insoluble in water, prone to oxidative decomposition, and not photothermal resistant, which limits their application. In the future, it is suggested that more research is carried out to improve the stability and durability of natural antibacterial agents, combining with natural polymer matrix to fabricate degradable, sustainable, and environmentally friendly food packaging to protect food from food pathogens. 

Inorganic antibacterial agents possess improved and novel biological characteristics because of their structure and develop with the nanotechnology advancements. The nanoparticles of metal and metal oxide are easier to process as well as having higher thermal stability [78]. Additionally, metal oxides offer an advantage by transporting important elements of minerals to the body. Antibacterial agents such as ZnO, magnesium oxide (MgO), TiO_2_, and metal oxide nanoparticles (ZnO-NPs, CuO-NPs, TiO_2_-NPs, SiO_2_-NPs, Al_2_O_3_-NPs, and Ag-NPs) have demonstrated successful antimicrobial activity against pathogens commonly found in food by generating reactive oxygen species (ROS), being biocompatible with cells and interacting with cell walls, or destroying the integrity of membrane [7,25,54], and these antibacterial agents have an advantage over organic antibacterial agents in terms of specificity and selectivity. Among the metal oxides, ZnO is one of the most broadly utilized materials in different fields because of its noteworthy antimicrobial and photocatalytic properties [24]. According to Yusof et al. [79], ZnO-NPs impact the antibacterial process by destroying the bacterial membrane due to having a mordant texture surface, which ultimately inhibits the growth of microorganisms. In recent years, thin films of Al-doped ZnO have been widely researched because they can alter the optical packaging properties [80]. Cu has the benefit of reduced toxicity in comparison to other metals because it is an important element for enzymes and metalloproteins [8]. CuO-NPs play a highly important role in active packaging as they possess wider spectrum effects of antimicrobials [81], and the process of growth inhibition of species by CuO-NPs was based on the size and concentration of nanoparticles. In addition, TiO_2_ has been recommended and approved for application in food packaging materials, cosmetics, and healthcare due to its low toxicity [82], and TiO_2_-NPs can reinforce compounds to offer huge mechanical strength and enable good antibacterial activity by invading the surface of bacteria and generating ROS [83,84,85]. Additionally, SiO_2_-NPs can be utilized as a contact surface for food packaging and for generating nonstick coatings for bags, bottles, and jars, thus preventing the spoilage of food items [81,86]. Aluminum oxide (Al_2_O_3_) coatings provide an alternative to metalized films as they are microwavable, transparent, biodegradable, light, and have effective barrier characteristics, so it has been suggested to utilize them in food packaging [87,88]. Al_2_O_3_ can be termed as ultrathin films (10–100 nm thickness) or nano-coatings with barrier properties against oxygen and water, along with the antimicrobial impacts [7,81,89]. However, Ag-NPs have been shown to have better bactericidal characteristics in comparison to metallic Ag or others against different pathogenic microorganisms such as viruses, bacteria, and fungi [7]. Pantic [90] found that the bactericide effect of Ag-NPs on *Salmonella choleraesuis*, *Listeria innocua*, *Bacillus cereus*, *Escherichia coli*, *Pseudomonas aeruginosa*, and *S. aureus* is achieved by increasing the toxic influence to the cells of bacteria. 

Additionally, organic synthetic polymers also play an important role in food packaging, including fermentation-based biopolymers (such as polylactic acid (PLA), polyhydroxyalkanoates (PHA), polyhydroxyvalerate (PHV), poly-b-hydroxyl butyrate (PHB), poly-3-hydroxybutyrate-co-3-hydroxyvalerate (PHBV) and exopolysaccharides (EPS), etc.), and bio-based biopolymers (such as bio-polyethylene (Bio-PE), bio-polyethylene terephthalate (Bio-PET), bio-polypropylene (Bio-PP), etc.) [91,92]. The fermentation-based biopolymers possess the function of antimicrobial activity after being modified or combining with bioactive compounds. Among them, PLA, PHA, and PHBV have a hopeful tomorrow for packaging applications which are biodegradable and with excellent gas barrier properties [93]. It should be noted that bio-PET, bio-PE, and bio-PP are also obtained from biological resources, but they are not biodegradable [92]. The applications of these materials in food packaging technology provide novel eco-friendly alternatives to petrochemical-based plastics, especially for single-use plastic goods, the object of the single-use plastics directive implementation of the new EU Commission’s European Green Deal of 2020 [92]. In the future, it is recommended to focus on the development of the correct combination of mixed materials, choosing proper additives to improve the functional properties of packaging materials, thus achieving better properties than petroleum-based plastics. 

### 2.5. Antimicrobial Bio-Nanocomposites

Bio-nanocomposites are a new generation of nano food packaging materials which are highly useful to inhibit the growth of microorganisms and further lead to the increase in the shelf life of food products and maintaining product quality and safety during transport, storage, and marketing. Bio-nanocomposites mainly comprise of bio-based polymeric matrices that are reinforced with nanofillers or nanoparticles, and they generally possess better characteristics (for example, antimicrobial, stable under high temperature and pressures) in comparison to traditional bio-based polymers [94]. Sharma et al. [2] and Zubair and Ullah [17] reported that bio-composites can be modified via fusion of nanofillers such as montmorillonite (MMT), Ag/AgO, ZnO/ZnO_2_, TiO_2_, and SiO_2_ and biodegradable polymers including PLA, polyhydroxylbutyrate (PHB), polybutylene succinate (PBS), polyvinyl alcohol (PVA), and poly-caprolactone (PCL), along with natural biopolymers such as polysaccharides, proteins, lipids, nucleic acids, etc. At present, there are various antimicrobial bio-nanocomposites that can inhibit the growth of microorganisms (pathogens and spoilage bacteria) on food surfaces and thus increase their shelf life, such as PLA/halloysite, PLA/Ag-NPs, hydroxyapatite/TiO_2_, layered silicate, chitosan/Ag-NPs, and cellulose nanocomposites. These versatile materials have good mechanical, thermal, and chemical resistance and barrier (oxygen, carbon dioxide, moisture, flavors, and grease), antimicrobial, and biodegradable properties [21,95,96,97], and they are also easy to process and have a low cost. Therefore, bio-nanocomposites have been considered as a promising alternative and a new generation of antimicrobial food packaging materials which are high-performance and eco-friendly have been used to inhibit the growth of contaminant microorganisms and further maintain food quality and safety during storage. 

According to the type of applied filler with antimicrobial properties, different types of antimicrobial bio-nanocomposites that are used in food packaging can be classified as follows: (ⅰ) clay and silicate-based bio-nanocomposites, (ⅱ) biopolymer-based bio-nanocomposites, (ⅲ) metallic-based bio-nanocomposites, (ⅳ) nano cellulose-based bio-nanocomposites, and (ⅴ) layered double hydroxide-based bio-nanocomposites. 

#### 2.5.1. Clay and Silicate-Based Antimicrobial Bio-Nanocomposites

To improve the functional properties of bio-based food packaging materials, they can be reinforced with silicate and clays (such as MMT, saponite, and hectorite). The addition of nanoclay Cloisite 30B (5.0% *w*/*w*) in PLA-based bio-nanocomposite films caused changes in the structural, mechanical, thermal, and antibacterial properties against microorganisms [98]. Kappa-carrageenan/locust bean gum blends with organically modified clays (Cloisite 30B) exhibited an inhibitory effect against L. monocytogenes [99]. The organoclay (Cloisite 30B)-based nanocomposites showed a strong bactericidal activity and beneficial bacteriostatic effects against Gram-positive bacteria and bacteriostatic activity against Gram-negative bacteria [100,101]. Rostamzad et al. [102] demonstrated that the water vapor permeability, mechanical strength, and tensile strength of bio-nanocomposite films were enhanced after fish myofibrillar protein (FMP) films were reinforced with MMT (1–5 wt.%) and microbial transglutaminase (1–3 wt.%). An epoxidized soybean oil-based nanocomposite was prepared with modification of organophilic MMT (clay incorporated in the oil-based polymer matrix), and the mechanical properties were improved [103]. Nanocomposites reinforced with 5% SiO_2_-NPs have better physical and mechanical properties, which acted as such because of the specific surface area and abrupt passage [104,105]. 

#### 2.5.2. Biopolymer-Based Antimicrobial Bio-Nanocomposites

Biopolymers are polymeric materials obtained from renewable biological resources (biomass, microorganisms, or biotechnology), but their commercial application is currently limited due to problems in their processing, performance, and cost [106]. However, the addition of nanofillers or nanoparticles in biopolymer-based packaging materials can improve their mechanical, thermal, antimicrobial, and barrier properties, due to the fact that their nanomaterials can interact with other matter at the atomic, molecular, or macromolecular level, thus affecting the functional behavior and practical use of biopolymer films. For example, CS-NPs in poly (vinyl alcohol)/bacterial cellulose film (CS-NPs/PVA/BC) showed a greater inhibition area of bacterial growth than PVA/BC film [107]. Furthermore, the CS-NPs can be modified to improve the antibacterial activity, such as grafting 4-pyridine carboxaldehyde [45]. Hosseini et al. [69] commendably fabricated fish gelatin/CS-NPs composite films for antimicrobial food packaging to extend the shelf life of products, and Trovatti et al. [108] used nano-fibrillated cellulose to make pullulan-based nanocomposite films showing properties in thermal stability, and the mechanical properties and tensile strength of the films were reinforced when compared to unfilled pullulan films. Polymer-based bio-nanocomposites PBS/ZnO exhibited antimicrobial activity against *S. aureus* and *E. coli* [109]. Production of WPI-based films with the reinforcement of cellulose nanofibers (C-NFs, 2, 4, 6% *w*/*w*) showed that films with 2% C-NFs had maximum mechanical strength, lower elongation, and higher stiffness [110]. 

Polymer bio-nanocomposites are novel materials characterized by biodegradable polymer reinforced with nanostructures causing changes in the structural, mechanical, thermal, and antibacterial properties against microorganisms [2,98]. Therefore, these bio-nanocomposites have promoted an increase in research related to polymer science and engineering. Their high performance, lightweight, and eco-friendly properties exhibit potential to replace traditional nonbiodegradable plastic packaging. Hence, it will promote the development of environmentally friendly food packaging material with a better shelf life and greater microbial resistance. 

#### 2.5.3. Metallic-Based Antimicrobial Bio-Nanocomposites

Metal-based antimicrobial bio-nanocomposites are made by incorporating metal NPs into polymeric films. They have successfully played an antibacterial role in nanocomposite antimicrobial systems due to the high specific surface area and elevated surface reactivity of the nano-sized antimicrobial metal/metal oxide particles enabling them to inactivate microorganisms more efficiently than their equivalents [111]. The commonly used metal and metal oxide nanomaterials are gold (Au), copper/copper oxide (Cu/CuO), Ag/AgO, Zn/ZnO, TiO_2_, alumina (Al_2_O_3_), and iron oxides (Fe_2_O_3_, Fe_3_O_4_). For instance, Al-Tayyar et al. [24] fabricated a novel antibacterial film (PVA/CS/ZnO-SiO_2_ bio-nanocomposite) which displayed superior antimicrobial activity to reduce the amounts of foodborne pathogens in packaged bread and greatly improved the visual appearance and shelf life. Hu et al. [112] loaded ZnO-NPs into chitosan/ZnO bio-nanocomposites films and found that the antimicrobial activity was enhanced. Addition of 2% CuO-NPs in nano-based agar films has demonstrated improved thermal stability and antibacterial properties, also with greater antioxidant activity and a greater barrier property of UV light [113]. 

**Figure 3:**
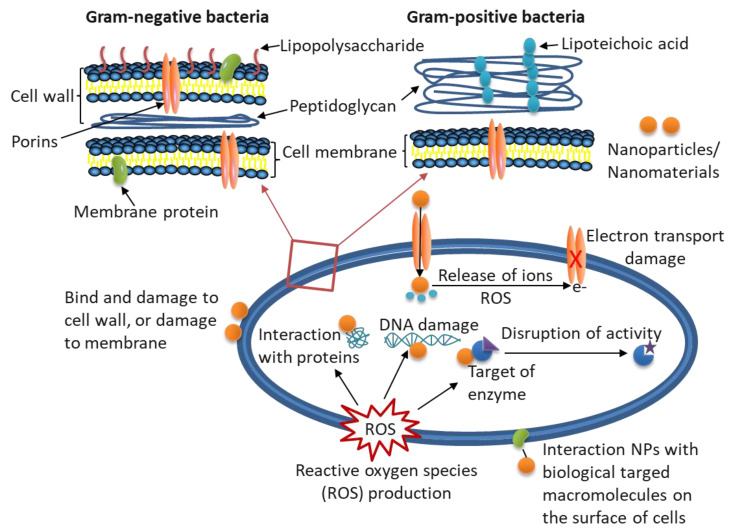
The potential antimicrobial mechanisms of nanoparticles/nanomaterials in food packaging (modified from Jamróz et al. [114]).

Currently, there are several mechanisms that can be used to explain the antimicrobial activity of these NPs, including interacting directly with the microbial cells, oxidizing cell components, producing secondary products, and dissolving heavy metal ions that cause damage [32,33,114], as shown in Figure 3, and many investigations have been performed on antimicrobial carriers, antimicrobial agents, growth inhibitors, and antimicrobial packaging films due to the nanoparticles or nanocomposite materials with antimicrobial activity. They have potential applications in food packaging, such as cheese, poultry, bread, fruits and vegetables, meat products, and fish products [2].

#### 2.5.4. Nano Cellulose-Based Antimicrobial Bio-Nanocomposites

In order to overcome the limitations of cellulose or nanocellulose in commercial application, nano cellulose-based bio-nanocomposites have attracted considerable attention from scientists. Cellulose fibrils and cellulose rich sources are two main materials for cellulosic nanofibers, and their preparation methods and properties were illustrated in Section 2.1. Abou-yousef et al. [1] studied the antibacterial activity of nanocomposites based on cellulose acetate/Cu-NPs; they were found to exhibit excellent antimicrobial activity against *S. aureus*, *P. aeruginosa*, *Candida albicans*, and *Aspergillus niger*, and the activity of cellulose acetate films with 2% Cu-NPs is higher than films with 6% Cu-NPs. Fortunati et al. [115] used melt extrusion to produce Ag NPs-cellulose nanocrystals/PLA-ontaining films. They presented antibacterial activity against Gram-positive and Gram-negative bacteria, and the presence of surfactant favored the tensile properties.

#### 2.5.5. Layered Double Hydroxide-Based Antimicrobial Bio-Nanocomposites

Layered double hydroxide (LDH) is a kind of layered inorganic solid. They can be modified by organic molecules and are gaining importance as nanofillers for the synthesis of polymer nanocomposites. LDH-based bio-nanocomposites have been demonstrated to enhance mechanical and barrier properties, hydrophilic surface properties, heat stability, flame retardancy, and antimicrobial activity [116]. In the study of Bugatti et al. [117], a Zn/Al-LDH composite embedded with 2,4-dichlorobenzoate and para-hydroxybenzoate exhibited bactericidal effects, and the release of antimicrobial moieties into the polymer was much faster than that of the molecular anions modified by the inorganic compound.

## 3. Application of Novel Bio-Based Antimicrobial Materials in Food Products

With the continuous improvement of living standards, food is required to have higher quality and longer shelf life. Food or beverage contamination and spoilage is always a concern in the food industry. In order to prevent foodborne pathogens and spoilage microorganisms, foodstuffs need suitable packaging during transport, storage, and marketing. Traditionally, the food is packaged with plastic that is extracted from non-renewable resources of fossil fuels; they are rarely recyclable and face hardship in disposal [2]. Therefore, the bio-based materials have become promising alternative materials since they are biodegradable, renewable, and eco-friendly [17]. At present, various novel bio-based antimicrobial materials have been used in food packaging, including biopolymer materials, nanoparticles/nanofillers, bio-based composites, and bio-nanocomposites. For instance, the polymer nanocomposite MMT of clay is a novel material for food packaging on the market, which can decrease the gas transmission rate resulting in keeping the freshness and prolonging the shelf life of oxygen-sensitive food products [118]. Sarojini et al. [73] studied the biological effects of mahua oil-based polyurethane/CS/ZnO-NPs nanocomposites for food packaging, which showed good anti-bacterial properties against Gram-positive and Gram-negative bacteria in carrot and improved the mechanical property. Ag-NPs have been used in fruit preservation due to their ability to catalyze the absorption and decomposition of ethylene emitted from fruit metabolism and their broad-spectrum inhibitory activities [2]. Badawy et al. [43] stated that CS-NPs-loaded monoterpenes exhibited high antibacterial effect on Salmonella typhimurium and E. coli in minced meat, and with antioxidant property thus extended the shelf life. In addition, bio-nanocomposites of moringa oil/CS-NPs-embedded gelatin nanofibers possessed high antibacterial activity against *L. monocytogenes* and *S. aureus*, and without any effect on the sensory quality of cheese [65]. Table 1 provides an overview of different types of novel bio-based antimicrobial materials in food packaging applications.

It should be noted that antibacterial bio-polymer is usually effective for some specific microorganisms, and not all microorganisms can degrade biopolymers. Antibacterial materials have their own antibacterial spectrum. The degradation of biodegradable polymers disposed in environments (e.g., landfills) is often induced by microorganisms (e.g., bacteria, fungi) via enzymatic catalysis processes. Furthermore, the polymer chains may also be broken down by nonenzymatic processes like chemical hydrolysis. This dilemma between biodegradable behavior and antimicrobial activity can be addressed well as long as the appropriate materials are selected in a targeted manner.

## 4. Regulatory and Safety Issues of Antimicrobial Nanomaterials

The safety and environmental impacts of different antimicrobial nanomaterials in food packaging must be identified to the fullest degree possible. Nanomaterials can migrate from packaging materials to the packaged foodstuffs and drinks and may pose risks to human health, animals, and the environment [134,135]. The generation of ROS by nanomaterials has been seen in both in vitro and in vivo conditions [136]. They may lead to generation of ROS in intestinal (or other) cells of humans after ingestion of food in which NPs have moved from the materials used to protect the food, causing cellular perturbation along with DNA damage and apoptosis. In addition, the presence of ROS in wounds may hamper wound healing [114]. Low ROS concentration will activate the signaling pathways, whereas its higher concentration can cause damage to mitochondria, cell membrane and other macromolecules [136]. ROS can also cause tissue degradation, which ultimately leads to carcinogenesis, aging, and other diseases [137]. Yet, the overall effects of nanomaterials on them are still not fully understood. Therefore, it is also highly recommended to study the migration path and pattern of NPs from nanocomposite packaging when in contact with food products and to establish an effective risk assessment system. Additionally, proper and validated procedures are lacking for all applications and characteristics; thus, safety decisions should be made with consideration of the nanoparticle size and the related safety evaluations, ultimately giving a clear answer as to whether and which nanomaterials can be a practicable substitute for traditional materials for food packaging application. Moreover, regulation and legislation still need a base to be developed.

## 5. Conclusions and Prospects

Traditional food packaging materials are extracted from non-renewable resources of fossil fuels (such as petroleum-based plastic materials) and they face difficulty in recycling and disposal. Recently, many types of bio-based antimicrobial materials have attracted considerable attention for packaging applications because of their unique chemical and physical properties, including natural antibacterial agents (e.g., nisin, ε-polylysine, enzymes, plant essential oils, tea polyphenols, etc.), inorganic antibacterial agents (some of the oxidized nanoparticles and metals), and organic synthetic antibacterial agents (such as allyl isothiocyanate and sorbic acids). The advanced coatings or films and biological additives are potentially feasible to help in preserving foodstuff. Meanwhile, nanomaterials have the capability to enhance thermal, mechanical, and antimicrobial properties and permeability as well as display other excellent functions and applications in food packaging. With the combination of biopolymer-based materials with nanofillers or nanoparticles, bio-nanocomposites can be obtained, which offers opportunities to create a sustainable and eco-friendly food packaging. Therefore, bio-nanocomposites are considered as a promising alternative and new generation of antimicrobial food packaging materials due to their biodegradability, high-performance, availability, and antimicrobial activity. They have the potential to be used as packaging materials for different food products, along with the manufacture of thermoformed containers, bottles for liquids, and sources for disposable applications. However, development of these excellent materials needs a multi-disciplinary research approach; producing cheaper materials and their successful implementation and commercialization have also become of utmost importance. Additionally, bio-based active, intelligent, and smart food packaging will be one strategy to address the global challenges of carbon neutrality. 

## Figures and Tables

**Table 1:** Different types of novel bio-based antimicrobial materials in food packaging.

Type of Materials	Food Product	Characteristics	References
Chitosan/carboxymethyl cellulose/ZnO-NPs	Egyptian soft white cheese	Antibacterial activity against Gram-positive (*S. aureus*), Gram-negative (*P. aeruginosa*, *E. coli*) bacteria and fungi (*C. albicans*); increasing the shelf life of white soft cheese.	[23]
Calcium alginate film loaded with ZnO-NPs	Ready-to-eat poultry meat	Antibacterial activity against *S. typhimurium* and *S. aureus*.	[119]
PLA/ZnO:Cu/Ag bio-nanocomposites	Food simulants (distilled water, 10% ethanol, and 3% acetic acid)	Good mechanical, thermal, and barrier properties to ultraviolet light, water vapor, oxygen, and carbon dioxide; antibacterial activity and low migration of nanoparticles into food simulants.	[120]
PVA/CS/ZnO-SiO_2_	Bread	Displayed superior antibacterial activity against Gram-positive bacteria (*S. aureus*, S33R) as well as Gram-negative bacteria (*E. coli*, IRAQ 3); greatly improved visual appearance of the bread, increase in shelf life.	[24]
ZnO-NPs/neem oil/CS	Carrot	The tensile strength, elongation, film thickness, and film transparency were improved; antibacterial activity against *E. coli*.	[121]
Mahua oil-based polyurethane/CS/nano ZnO composite film	Carrot	Improved the mechanical property and reduced the permeability of oxygen and moisture; excellent anti-bacterial properties against Gram-positive and Gram-negative bacteria, reducing bacterial contamination; enhances the shelf life of carrot.	[73]
CS/PVA/TiO_2_ bio-nanocomposite	Soft white cheese	Effective antibacterial activity against Gram-positive (*S. aureus*) and Gram-negative (*P. aeruginosa*, *E. coli*) bacteria and fungi (*C. albicans*); extension in shelf life.	[122]
Cellulose acetate (CA)/Cu-NPs	Food simulants	Antibacterial activity against *S. aureus*, *P. aeruginosa*, *C. albicans*, and *A. niger*; CA film with 6% exhibited lower activity than film with 2% Cu-NPs; enhancement of thermal stability.	[123]
Poly(glycidyl methacrylate covinyl ferrocene); graphene oxide/Fe_3_O_4_-NPs	Fish	An effective platform to produce reliable xanthine biosensor; fish meat freshness control.	[124]
CS-NPs-loaded monoterpenes	Minced meat	Exhibited good in vivo antimicrobial (against *S. typhimurium* and *E. coli*) and antioxidant property; extension in shelf life.	[43]
Corn starch/talc NPs	Tomato	Improvement in strength, tightness, and barrier properties; reduction in water vapor and oxygen permeability.	[125]
ε-Poly-lysine/CS-nanofibers	Chicken	Exhibited antibacterial effect on *S. typhimurium* and *S. enteritidis* on chicken; increasing the shelf life and maintaining the quality of the packed food.	[47]
Moringa oil/CS-NPs-embedded gelatin nanofibers	Cheese	Possessed high antibacterial activity against *L. monocytogenes* and *S. aureus*, without any effect on the sensory quality of cheese.	[65]
Pectin/ LDH-salicylate	Fresh apricot	Improved elongation at break point for pectin; improved water vapor barrier properties; extension in shelf life.	[126]
Starch nanocomposite films containing nanoclay (halloysite) and nisin	Soft cheese	Mechanical properties were improved with halloysite addition; antimicrobial activity against *L. monocytogenes*, *Clostridium perfringens*, and *S. aureus*.	[127]
Quaternary ammonium salt-modified CS and PVA	Strawberries	Highly efficient antifogging and antibacterial activity (against *E. coli*, *S. aureus*, and *Botrytis cinerea*).	[42]
Thyme EOs and MMT based sweet potato starch films	Baby spinach leaves	Antibacterial activity against *E. coli* and *S. Typhi* on fresh baby spinach leaves; extension in shelf life.	[128]
Addition of MMT to WPI matrix	Food simulants (water, 3% acetic acid, 15% ethanol, olive oil)	Increased tensile strength of the WPI film, and swelling of the WPI film was reduced.	[129]
Alginate/nano-Ag coating	Shiitake mushroom (*Lentinus edodes*)	Reduced mesophilic, pseudomonad, yeasts and molds counts; spoilage reduction, improvement of sensory attributes, lower weight loss.	[130]
Shirazi balangu seed mucilage edible coating	Beef slices	Enhancement of the shelf life of beef by preventing lipid oxidation and microbial spoilage.	[131]
Tilapia skin gelatin incorporated with ethanolic extract from coconut husk/Cloisite Na^+^ nanoclay	Meat powder	Lower lipid oxidation products; improved moisture barrier properties; extension in shelf life.	[132]
Nanoemulsion coating of CS/mandarin EOs	Green beans	Antimicrobial activity in *L. monocytogenes*; exhibited a slight antagonistic effect and had a slight detrimental impact on color properties when combined with pulsed light, but promising with high hydrostatic pressure.	[133]

## Data Availability

All data is publicly available.
